# Social, political, commercial, and corporate determinants of rural health equity in Canada: an integrated framework

**DOI:** 10.17269/s41997-022-00630-y

**Published:** 2022-04-18

**Authors:** Betsy Leimbigler, Eric Ping Hung Li, Kathy L. Rush, Cherisse Lynn Seaton

**Affiliations:** University of British Columbia - Okanagan Campus, Kelowna, BC Canada

**Keywords:** Rural health equity, Integrated framework, Determinants of health, Équité en santé rurale, cadre intégré, déterminants de la santé

## Abstract

People in rural and remote areas often experience greater vulnerability and higher health-related risks as a result of complex issues that include limited access to affordable health services and programs. During disruptive events, rural populations face unique barriers and challenges due to their remoteness and limited access to resources, including digital technologies. While social determinants of health have been highlighted as a tool to understand how health is impacted by various social factors, it is crucial to create a holistic framework to fully understand rural health equity. In this commentary, we propose an integrated framework that connects the social determinants of health (SDOH), the political determinants of health (PDOH), the commercial determinants of health (ComDOH), and the corporate determinants of health (CorpDOH) to address health inequity in rural and remote communities in Canada. The goal of this commentary is to situate these four determinants of health as key to inform policy-makers and practitioners for future development of rural health equity policies and programs in Canada.

## Résumé

Les personnes vivant dans les régions rurales et éloignées sont souvent plus vulnérables et présentent des risques plus élevés pour la santé en raison de plusieurs défis incluant l’accès limité aux services et programmes de santé abordables. Lors des événements perturbateurs, les populations rurales font face à des obstacles et défis uniques en raison de leur éloignement et de leur accès limité aux technologies digitales. Alors que les déterminants sociaux de la santé sont un outil pour comprendre l’effet de divers facteurs sociaux sur la santé, il est crucial de créer un cadre holistique pour bien comprendre l’équité en santé rurale. Dans ce commentaire, nous proposons un cadre intégré qui relie les déterminants sociaux de la santé (SDOH), les déterminants politiques de la santé (PDOH), les déterminants commerciaux de la santé (ComDOH) et les déterminants de la santé des entreprises/corporatifs (CorpDOH) pour lutter contre les inégalités en santé dans les régions rurales et éloignées au Canada. Le but de ce commentaire est de situer les déterminants sociaux, politiques, commerciaux et corporatifs de la santé comme étant essentiels aux responsables de la formulation des politiques publiques ainsi qu’aux clinicien(ne)s dans l’avenir des politiques et programmes d’équité en santé rurale au Canada.

## Introduction

Health is not just confined to the medical realm; it is also fundamentally social, political, organizational, and commercial. Rural health inequities are often seen as outcomes of systemic differences in health that are caused by the unfair distribution of resources, wealth, and power in society (Commission of the Pan American Health Organization on Equity and Inequalities in the Americas, 2019). In Canada, the majority of healthcare practitioners and policy-makers consider the social determinants of health (SDOH) as a foundational framework to promote equity. SDOH include “the conditions in which people are born, grow, live, work and age” (WHO, 2021). However, SDOH alone do not adequately recognize the disparities and inequities that persist in rural and remote regions.

In the past decade, researchers have begun to explore the political, commercial, and corporate determinants of health (e.g. Dawes, 2020; Kickbusch, 2015; Millar, 2013). Policy-makers and corporate stakeholders in legal and regulatory systems often determine, if not dictate, the design and development of the market system and health service programs that impact the health and well-being of citizens. However, in Canada, many health-related strategic plans and policies are based on “urban-centric”, cost-effective, and efficiency-driven planning frameworks which often ignore the needs and unique challenges faced by rural and remote communities. Similarly, stakeholders with corporate and commercial interests have often viewed rural markets as unattractive and not profitable, further escalating the vulnerability and disadvantages of the population.

Although SDOH have received wide acknowledgement and discussion among policymakers and practitioners, less attention has been given to the political, commercial, and corporate determinants of health in the Canadian context (Kickbusch, 2015; Dawes, 2020; Millar, 2013). Health inequities in rural and remote areas matter for reasons of access, equity, and inclusion. Issues include lack of access, ranging from access to physicians to access to drinking water on many First Nations reserves^1^. Additionally, legacies of exclusionary policy that have created and sustained health inequities can be traced back to political decisions that intersect with commercial and corporate determinants. We argue that an integrated determinants of health framework is inextricable from understandings of equity and inclusion, and such a framework will better serve rural and remote areas of the country.

## The social, political, commercial, and corporate determinants of rural health equity

According to Statistics Canada, around 17.8% of the Canadian population live in rural areas (Statistics Canada, 2022). Compared to their counterparts in urban settings, rural and remote residents can face unique barriers such as lack of access to higher education, job opportunities, technologies and technology infrastructure (i.e. high-speed internet), comprehensive health services and goods, public funding and resources for sustainable community development. All these disparities are a sum of political, market, and socio-cultural influences over a long period of time.

In Canada, health authorities and practitioners have adopted SDOH in designing and reconfiguring health care services and programs. Previous studies have pointed out that the SDOH have a strong connection to governance and public policy (Manzano & Raphael, 2010). This highlights the central and important role that political structures play in social determinants of health.

In the past decades, new laws, policies, and government programs have been established to increase access to health care and treatments. Kickbusch (2015) notes the importance of analyzing “…how different power constellations, institutions, processes, interests, and ideological positions affect health within different political systems and cultures and at different levels of governance” (Kickbusch, 2015, p. 1). In his seminal work on the political determinants of health (PDOH), Dawes (2020) argues for the gravity of combining an understanding of political structures with a critical analysis of health equity actors involved in the creation of new laws in the United States such as the Affordable Care Act. Dawes (2020) examines political actors’ roles in ensuring health equity, and PDOH can refer to every power structure and political decision that creates social structures that impact peoples’ health. PDOH can also refer to access and equity, and even expand to government or partisan decisions that are made or not made regarding resource allocation (e.g. vaccination) as well as coalitions, political actors, and equity advocates.

Closely connected to governance but distinctly different are commercial determinants of health (ComDOH), corporate determinants of health (CorpDOH), and their interconnection with social and political determinants of health that have been a growing focus for scholars (Millar, 2013). The term “commercial determinants of health” initially referred to the recognition of market and non-market activities of harmful commodities (e.g. tobacco, alcohol) as drivers of non-communicable diseases (Kickbusch et al., 2016; Moodie et al., 2013). Some would consider these “lifestyle diseases” (Freudenberg, 2012), “industrial epidemics”, or “profit- or corporate-driven diseases” (Gilmore et al., 2011; Jahiel, 2008) given the prominent involvement of market agents and commercialized goods. However, determinants of health are not limited to the production and consumption of harmful or healthful commodities in the market. Millar (2013) introduced the concept of “corporate determinants of health”, highlighting corporate and organizations’ roles in the health economy. He further connected the new concept with corporate social responsibility and moved beyond the transaction-based discussion (i.e. seller–buyer relationship). For Millar, CorpDOH included employee well-being and the triple bottom line (profit, people, and the planet).

Multiple determinants of health frameworks have been proposed in the past decade, and we observe that these existing frameworks have not addressed the specific health needs and service gaps in rural and remote communities. We therefore conceptualize an integrated determinants of health framework that includes and combines the political, commercial, and corporate determinants with the World Health Organization’s (2021) list of SDOH. This is essential for understanding rural health challenges in rural and remote Canada as outlined in Table 1.

**Table 1:** Definitions of social, political, corporate, and commercial determinants of health and their relevance and application to rural communities

	Definition	Relevance and application to rural communities
Social determinants of health (SDOH)	“The conditions in which people are born, grow, live, work and age” (WHO, 2021).	• Jobs in rural communities are uniquely different from types of jobs in urban centres. • Particularly during the COVID-19 pandemic, people in rural and remote areas experienced poorer internet connectivity, impacting ability to access services. • Geography plays a role in extreme weather events and their impact on rural communities. • Cultural elements are core to rural ways of life and health. • Factors such as car ownership and type of employment are significant for health outcomes. Public transportation in rural and remote areas is limited and impacts ability to reach health providers.
Political determinants of health (PDOH)	The levels of governance, systems, institutions, and political decisions that impact peoples’ health (Kickbusch, 2015; Dawes, 2020)	• Can include coalitions between organizations, health equity groups and their lobbying efforts, voter engagement and turnout, and other political actors. • Rural and remote communities have their own governance structures, and lack of structures creates greater challenges in advocating for needs. This also relates to areas such as communication, transportation, infrastructure, and links to commercial determinants of health. Such barriers impact lifestyles. • Funding and political will to address health concerns in rural Canada. Rural and remote communities tend to have worse health outcomes than urban centres. The reasons for this are multi-fold but political decisions that impact building, funding, and health policy are significant in health outcomes. • Urban-centric policy design is another way that health in rural areas is politically determined.
Commercial determinants of health (ComDOH)	Recognition of market and non-market activities of harmful commodities (e.g. tobacco, alcohol) as drivers of non-communicable diseases; marketing, lobbying of corporations (Kickbusch et al., 2016; Moodie et al., 2013).	• Commercial determinants broadly refer to commodities, advertising, and commercialization of products, goods, and services. • In many rural communities, food is grown locally and hunting is part of a way of life. This intersects with the cultural elements of the SDOH unique to rural ways of life. • There is often a lack of big box stores in smaller communities, to avoid competition and to value and promote the presence of small family businesses. • Significantly higher cost of food in many Northern as well as rural and remote communities is a key example of commercial determinants of health.
Corporate determinants of health (CorpDOH)	Corporate and organizations’ roles in the health economy (Millar, 2013; McKee & Stuckler, 2018).	• Can refer to intellectual property protection of medicines, soda taxes, and specific corporations or industries working in rural and remote settings. • Types of industries that are drawn to rural areas in Canada include agriculture, mining, and oil industries. There is significant intersection between corporate and political determinants of health. • Presence of economic “ghost towns” based on certain industries and their withdrawal. • Corporate refers to global corporations as well as specific corporations or firms. • Urban-centric policy results in large stores built closer to denser urban areas, resulting in a different economic and corporate landscape in rural Canada.

## Determinants of rural health equity

**Fig. 1:**
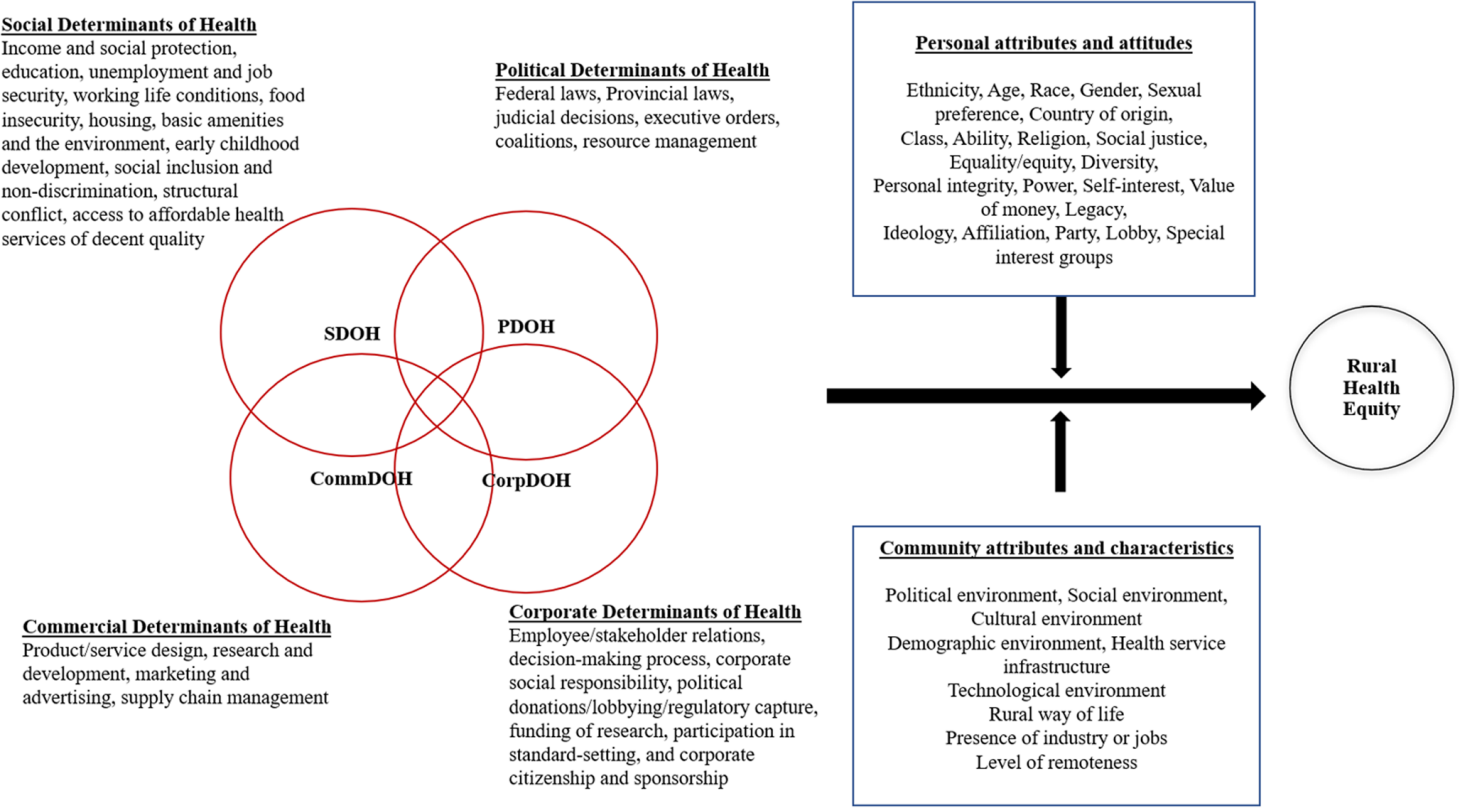
Integrated determinants of health framework for rural health equity

Rural populations in Canada currently experience a unique set of challenges, with many communities historically disadvantaged due to age, geography, experiences with political systems, and challenges faced by rural and remote Indigenous communities in particular (Wilson et al., 2020). Rural settings can create conditions that exacerbate disparities for ethnic and racial minorities. The proposed integrated determinants of health framework (Fig. 1) allows for inter- and intradisciplinary work that systematically combines aspects of social, political, corporate, and commercial determinants. Centering health equity actors as well as corporate actors who are involved in policymaking processes in Canada, particularly in rural Canada, can help address issues in rural and remote areas. Figure 1 illustrates the ways in which the four sets of determinants of health are connected and overlap. The “personal” and “community” attributes illustrate how rural health equity, or the conditions under which individuals have access to resources that create opportunities for health, is impacted by these factors, as well as the four determinants.

Based on the integrated determinants of health framework, we propose four sets of strategic recommendations to inform policy-makers, practitioners, corporate stakeholders, and community leaders to continue working towards health equity in rural Canada (Table 2). For instance, it is important to diligently develop a rural-oriented health care and market system to ensure rural populations’ equitable access to health services and healthy goods. Creating inclusive and respectful intra- and inter-community networks with key stakeholders and organizations is critical to address health and health care–related challenges. Collaboration and creativity are crucial to build capacity. We recommend that community stakeholders and policy-makers develop an ecosystem to embrace collaboration and innovation. It is important to create a sustainable platform to empower and engage community stakeholders to create adaptive solutions to address opportunities and challenges unique to rural and remote communities.

**Table 2:** Strategic recommendations for implementing the integrated determinants of health framework

Focus	Strategic recommendations
Intra- and inter-community networks	Develop an inclusive and diverse network to identify and co-define barriers faced by rural and remote communities in Canada.
Multidisciplinary and multisectoral collaboration	Leverage resources and capabilities through multisectoral and multidisciplinary collaboration in policy-making, research and development, program implementation, and impact assessment.
Rural-oriented frameworks	Mobilize corporate and health policy-makers to move beyond the “urban-centric” and “cost-benefit” mentalities and co-create a resilient and sustainable “rural-oriented” framework for residents in rural and remote Canada.
Innovation	Explore new forms of governance, organizations (e.g. social enterprise), platforms, and innovation (e.g. social innovation) to address rural health equity–related challenges.

These recommendations provide an early attempt to strategically adopt the integrated framework to develop a more equitable, inclusive, and adaptive determinants of health framework to address rural health inequity and disparity. Rural health inequities are avoidable, preventable, and unjust. We believe that the integrated determinants of health offer a new blueprint for key stakeholders to make important inroads in managing sustainable systems for health equity in rural and remote Canada.

## Conclusion

Inequities and systemic disparities among rural and remote-living residents persist in Canada. Challenges faced by rural and remote communities have been amplified due to new public health protocols and many other socio-political issues. An urgent focus on rural health equity is imperative, with growing recognition that urban-centric policy is not translatable to many areas of Canada, and that comprehensive frameworks are essential in combating health inequity.

The proposed integrated framework illustrates how these complementary sets of determinants encompass health equity principles at their core, and provides a tool to understand the social, political, commercial, and corporate structures that have created our health systems and impact health in rural areas of Canada. The integrated determinants of health framework can be used by public health scholars, political scientists, and interdisciplinary researchers to centre health equity and to highlight the actors and processes involved in rural health.

## Footnotes

1 The authors acknowledge the diverse definitions of “rural” and “remote” (Subedi et al., 2020), as well as the intersectionality of barriers to health equity in rural First Nations communities.
